# Tissue-Based MicroRNAs as Predictors of Biochemical Recurrence after Radical Prostatectomy: What Can We Learn from Past Studies?

**DOI:** 10.3390/ijms18102023

**Published:** 2017-09-21

**Authors:** Zhongwei Zhao, Carsten Stephan, Sabine Weickmann, Monika Jung, Glen Kristiansen, Klaus Jung

**Affiliations:** 1 Department of Urology, University Hospital Charité, 10117 Berlin, Germany; zhongwei.zhao@charite.de (Z.Z.); carsten.stephan@charite.de (C.S.); Sabine.weickmann@charite.de (S.W.); monika.jung@charite.de (M.J.); 2 Berlin Institute for Urologic Research at the Charité, 10115 Berlin, Germany; 3 Institute for Pathology, University Hospital Bonn, 53123 Bonn, Germany; glen.kristiansen@ukbonn.de

**Keywords:** microRNA, prognostic biomarkers, prostate cancer, radical prostatectomy, biochemical recurrence

## Abstract

With the increasing understanding of the molecular mechanism of the microRNAs (miRNAs) in prostate cancer (PCa), the predictive potential of miRNAs has received more attention by clinicians and laboratory scientists. Compared with the traditional prognostic tools based on clinicopathological variables, including the prostate-specific antigen, miRNAs may be helpful novel molecular biomarkers of biochemical recurrence for a more accurate risk stratification of PCa patients after radical prostatectomy and may contribute to personalized treatment. Tissue samples from prostatectomy specimens are easily available for miRNA isolation. Numerous studies from different countries have investigated the role of tissue-miRNAs as independent predictors of disease recurrence, either alone or in combination with other clinicopathological factors. For this purpose, a PubMed search was performed for articles published between 2008 and 2017. We compiled a profile of dysregulated miRNAs as potential predictors of biochemical recurrence and discussed their current clinical relevance. Because of differences in analytics, insufficient power and the heterogeneity of studies, and different statistical evaluation methods, limited consistency in results was obvious. Prospective multi-institutional studies with larger sample sizes, harmonized analytics, well-structured external validations, and reasonable study designs are necessary to assess the real prognostic information of miRNAs, in combination with conventional clinicopathological factors, as predictors of biochemical recurrence.

## 1. Introduction

Prostate cancer (PCa) is the second most common cancer among men worldwide. It encompasses 15% of all diagnosed male malignancies every year, with an estimated 1.112 million new cases and 307,000 deaths according to the last global cancer statistics in 2012 [1].

Since the mid 1980s, the widespread use of the prostate-specific antigen (PSA) has substantially shaped the management of this cancer, but its overdiagnosis and overtreatment has gained increasing attention after a controversial debate on the PSA-based early detection and screening approach [2,3]. This is a result of the heterogeneous behavior of the disease from the entirely indolent to the extremely aggressive tumor. Numerous pre- and post-treatment nomograms based on well-established clinicopathological factors, such as clinical and pathological tumor stage, bioptic and pathological histological grading systems according to Gleason, and PSA values, have been used to estimate the individual risk of the disease course. This particularly refers to the prediction of different clinical end points like biochemical recurrence (BCR), occurrence of metastases, cancer-specific death, and overall survival [4,5,6]. However, the achieved accuracy of the outcome prediction using these nomograms is generally limited, resulting in an agreement between predicted and observed outcomes of only 70–80%. Thus, the identification of prognostic factors and the elucidation of the underlying molecular mechanisms that determine the course of the disease are essential future tasks for improving the cancer decision-making process [7,8]. This is true both for the risk estimation after PCa detection and for the follow-up after treatment.

Radical prostatectomy is the surgically preferred treatment option with curative intention of clinically significant PCa. Molecular markers of genomic, transcriptomic, proteomic or metabolomic nature are capable of enhancing the prediction accuracy if they are included in prediction tools that are based on only clinicopathological factors [9,10,11,12]. For such an approach, microRNAs (miRNAs), as decisive regulators of the cellular processes, are also candidate biomarkers [13,14,15]. miRNAs can function both as tumor suppressors or oncogenes in urological tumors as described in several recent reviews [16,17,18,19,20,21]. More detailed information regarding their special role in cancerogenesis and the progression of these tumors as well as their biogenesis and general function can be found in these reviews.

The expression of miRNAs can be specifically quantified in prostatectomy tissue samples. These analytes have been suggested in previous studies as promising prognostic markers to improve the prediction of the biochemical recurrence of PSA as the first alarming sign of cancer relapse after prostatectomy [22,23,24,25]. Approximately 15–30% of patients suffer from a biochemical recurrence after radical prostatectomy [26,27]. Thus, an early and reliable detection of these PCa patients at risk after radical prostatectomy would improve the decision-making for the initiation of adjuvant therapy and for the selection of patients who need a more frequent monitoring during follow-up.

Therefore, in the present review, we aimed (a) to compile the relevant data of existing miRNA-based studies, (b) to identify the most promising miRNAs as potential predictors of biochemical recurrence proven in several independent studies, (c) to critically assess the real benefit of these new markers compared or in combination with the conventional parameters and (d) to formulate preconditions for robust assays to translate validated results into clinical practice.

## 2. Literature Search Strategy

### 2.1. Medical Subject Heading (MeSH) Terms and Keywords

For this review, a PubMed search was performed for original articles in the database from 2008 to May 2017. The search strategy included the followings terms: the MeSH term “MicroRNAs” combined with the search string [“microRNAs” OR “microRNA” OR “micro-RNA” OR “micro-RNAs” OR “miRNAs”], the MeSH term “prostatic neoplasms” linked with the keyword “prostate cancer” using the Boolean operator “OR” and always connected with the search strings [“biochemical recurrence” OR “recurrence” OR “biochemical relapse” OR “biochemical failure”], and “radical prostatectomy” using the Boolean operator AND. Furthermore, references in the identified articles and reviews were considered to detect additional relevant articles. Publications were included in this review only if (a) they were peer-reviewed and supplied with full text in English, (b) the sample resources were tissue specimens, either fresh-frozen or formalin-fixed, paraffin-embedded (FFPE) tissue, whereas articles pertaining to miRNAs from blood, urine, cell lines, and animal models were disregarded, and (c) study objects were single miRNAs, patterns of various miRNAs, or miRNAs combined with clinicopathological variables, resulting in potential prognostic value for BCR. 

### 2.2. Defining BCR as the Clinical Endpoint

BCR refers to the occurrence of increasing PSA value after its decline due to treatment. Radical prostatectomy and radiotherapy are the two main curative options for treatment of prostate cancer. Here, we exclusively refer to the surgical option as only this treatment mode allows the investigation of tissue samples of the removed cancer.

After radical prostatectomy, circulating PSA rapidly declines in a biphasic elimination, with a half-life of approximately one to three days [28]. Thus, patients with a preoperative PSA value of 20 µg/L generally reach a PSA level of <0.1 µg/L after 10 to 20 days, but not later than four weeks after successful operation [28]. According to the guidelines of the European Association of Urology (EAU), a PSA value <0.1 µg/L after radical prostatectomy is considered as undetectable. In this case, the definition of BCR is based on a renewed PSA increase to >0.2 µg/L that is confirmed by two consecutive elevated values [29]. Patients with increasing PSA values before the PSA nadir is reached should not be included in this biochemical recurrence group as the clinical outcome of patients with such a persistent PSA value after radical prostatectomy is generally poor. The 0.2 µg/L PSA cutoff also corresponds to the definition of BCR recommended by the American Urological Association (AUA) Prostate Guideline Update Panel [30]. However, it should be pointed out that this panel registered in their literature search of 145 studies more than 53 varying different definitions of biochemical recurrence after radical prostatectomy. The improved detection limit and analytical accuracy to measure low PSA values contributed to the recommendation to use the 0.2 µg/L PSA cutoff instead of 0.4 µg/L as previously suggested [31].

Thus, various factors summarized in Table 1 influence BCR directly, such as adverse tumor characteristics, or indirectly, such as different PSA analytics, varying definitions of BCR, and the clinician’s judgment of BCR. Within a selected definition of BCR, the tumor characteristics of the individual patient are the most important factor that determine the occurrence of BCR [5,6,32,33,34]. The numerous pre- and postoperative nomograms predicting BCR-free probability after radical prostatectomy are based on these clinicopathological data [4,5,6,35,36]. Thus, the clinical usability of all additional classifiers, in our case miRNAs, as potential more informative decision-making tools or adjunctive parameters have to be validated in relation to these conventional clinicopathological data in multivariate statistical models. Only their additional diagnostic benefit or cost-efficiency in comparison to conventional tools would justify the introduction in clinical practice. In the present review, we focused on the assessment of this aspect in the studies. Despite the controversial discussions regarding a standardized definition of the PSA cutoff of BCR and its use as a surrogate for the clinical outcome in these patients [37,38,39,40], an increasing PSA concentration after radical prostatectomy is considered by the clinician to be the first sign of potential later cancer metastasis [41]. It is obvious that BCR is not equal to clinical relapse, but elevated postoperative serum PSA levels enable the isolation of patients with high risk of true disease recurrence [41]. Therefore, in our tabulated summary reports, we included the specific cutoffs of BCR used in the particular studies.

## 3. Overview of the Evaluated Studies

### 3.1. Number of Annual Publications and Type of Tissue Samples Used in the Studies

**Figure 1:**
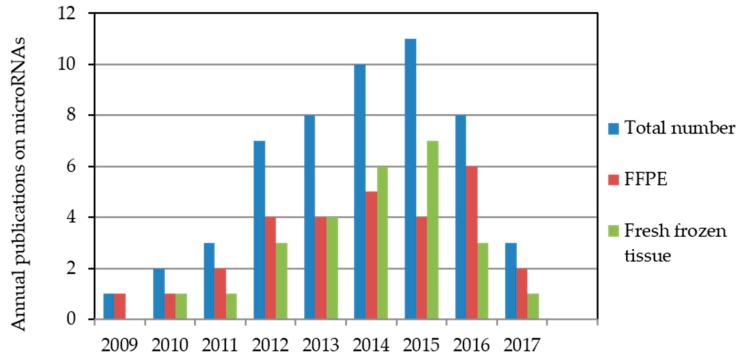
Annual microRNA publications indexed in the PubMed database relating to biochemical recurrence after radical prostatectomy. The literature search was performed for the period from October 2008 to May 2017 with miRNA measurements in formalin-fixed, paraffin-embedded (FFPE) or fresh-frozen tissue samples. Two studies used both FFPE and fresh-frozen tissue samples [49,50].

After preliminary screening of 148 papers, we identified 53 publications that complied with the described inclusion criteria. Forty-nine of these 53 articles were published in the past seven years. Only three papers appeared before 2011 [22,23,24]. Details can be seen in Figure 1.

Interest in the prognostic value of miRNAs in PCa has been reflected in the increasing number of publications. In 2009, Tong et al. [22] presented the first relevant study on the prognostic potential of miRNAs in PCa tissue; miR-135b and miR-194 were proven to reflect a tendency for early PCa relapse by comparing patients with early and late BCR. The results summarized in this review of 53 studies are based on data from 29 and 26 studies that analyzed FFPE and fresh-frozen tissue samples, respectively. In two studies, both FFPE and fresh-frozen tissue samples were used [49,50].

### 3.2. Characteristics of the Studies Evaluated in This Review

Biomarker studies with the intention to develop a robust assay for clinical practice must successfully undergo various phases of testing. Simply speaking, a discovery phase with the identification or selection of potential candidate biomarkers based on different principles for the intended objective should be distinguished from validation processes [51]. This classification with their subdivided characteristics is helpful to facilitate the assessment of studies and has therefore been adapted with regard to the use of miRNAs as BCR biomarkers in Table 2. On this basis, essential data and results of every study of the 53 evaluated studies including our own assessment have been compiled in Table 3. For the sake of clarity and facilitating the later discussion, the studies are listed by year of publication and are numbered accordingly.

#### 3.2.1. Dysregulated miRNAs with Association to Biochemical Recurrence

**Figure 2:**
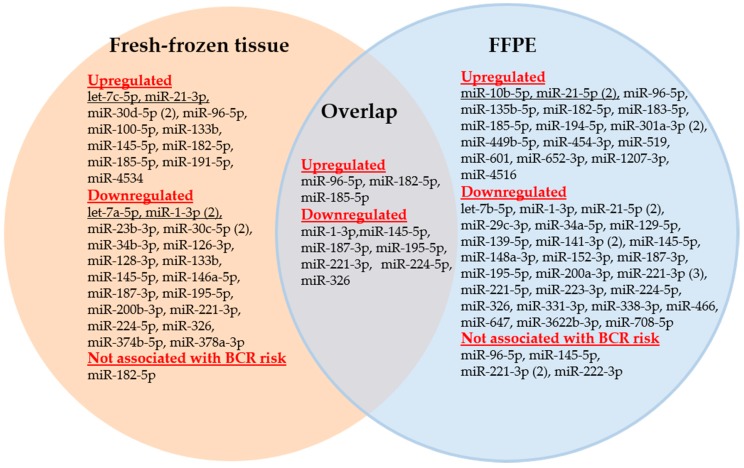
Venn diagram of the miRNAs analyzed in FFPE and fresh-frozen tissue samples of studies examining the predictive capability of miRNAs for biochemical recurrence. Numbers in parentheses indicate the number of studies that examined the respective miRNA.

The differentially expressed miRNAs in prostatectomy tissue samples that have been proven to be potentially predictive BCR markers in the 53 evaluated studies are represented in Figure 2 as a Venn diagram.

As previously mentioned, both samples from fresh-frozen tissue and FFPE archived tissue blocks were used for analysis of miRNAs in these studies. In contrast to fresh-frozen tissue, FFPE tissue samples are easily available as they are generally used in the tissue-based diagnostic routine process and do not require time-consuming workflow in comparison to fresh-frozen tissue samples. FFPE blocks are archived in repositories of the pathological institutes along with all clinical and pathological information. In contrast to the non-stability of mRNAs in FFPE tissue, miRNAs were found to be congruently expressed in fresh-frozen and FFPE tissue samples including prostate cancer [108,109,110,111]. Because of their small size and association with protectively acting macromolecules, miRNAs are obviously more robust molecules and are less affected by degradation processes than mRNAs. This was also demonstrated in model experiments of RNA degradation [112]. Li et al. [113] showed comparable miRNA profiles between FFPE and paired snap-frozen materials with R^2^ > 0.95. Moreover, this observation is consistent with the results of Casanova-Salas et al. [49] and Kristensen et al. [50], who used both FFPE and fresh-frozen tissue samples in their BCR studies (see Table 3, Study nos. 22 and 46). However, there are conflicting data on the stability of miRNAs in FFPE tissue blocks stored for more than ten years [108,109,111,114]. Two studies recently proved the differential long-term stability of various miRNAs in FFPE samples over ten years [114,115], probably depending on the different GC contents in the distinct miRNAs [115]. This issue needs to be controlled in studies using long-term archived samples to consider this possible storage effect for a correct assessment of analytical data [114].

In reviewing the 53 studies, 41 distinct miRNAs were described in FFPE and 27 miRNAs in fresh-frozen tissue samples as significant miRNAs (Figure 2). Moreover, only 10 miRNAs were simultaneously detected in both sample types as shown in the overlap section of Figure 2. As miR-21-5p, miR-133b, and miR-145-5p were found to be both up- and downregulated in various studies, a total of 58 distinct miRNAs were used as potential BCR markers. Of these 58 miRNAs, only 15 miRNAs were examined in at least two studies, whereas 43 miRNAs were determined in only one study (Table 4, Table S2). The direction of the dysregulation of the miRNAs is indicated by arrows in Table 4.

#### 3.2.2. miR-221-3p, miR-21-5p, miR-145-5p, miR-1-3p, and miR-96-5p, the Most Frequently Analyzed miRNA-Based BCR Markers

The miRNAs miR-221-3p, miR-21-5p, miR-145-5p, miR-1-3p, and miR-96-5p were found to be the most frequently analyzed miRNAs in the reviewed BCR studies. Their results are of particular interest as they allow some general conclusions with regard to the potential predictive BCR capability of miRNAs but also to future research requirements. In referring to the direction of the dysregulation of these miRNAs in the corresponding studies as indicated in Table 4 and subsequently mentioned using the list number from Table 3, the following short comments should summarize the situation.

- **miR-221-3p.** Three of the four studies confirmed the downregulated expression of miR-221 as a useful BCR predictor and independent factor in multivariate analyses with the standard clinicopathological variables (Study nos. 3, 31, and 46; [24,50,83]). Kristensen et al. [50] (Study 46) validated miR-221 in two independent BCR cohorts and an additional external validation using a publicly available data set as part of their 3-miRNA signature while Spahn et al. [24] (Study 3) proved the usefulness of this miRNA especially in high-risk PCa patients. Thus, these studies can be assessed as successful approaches from the discovery phase to validation by clinical assessment with the aim to develop a potential clinical tool as suggested in Table 1. The miRNA tool miQ that was primarily developed for diagnostic purposes included the also downregulated 5p strand of miR-221 in predicting BCR (Study 17, [67]). Strong correlations were observed in these studies between the increased expression of miR-221 and the tumor stage, Gleason score, and the pre-operative PSA level. In contrast, these correlations were not found in Study 9 with the missing predictor evidence of miR-221 [58]. However, this failure could also be caused by the short follow-up period of less than two years in this study.
- **miR-21-5p.** Increased and decreased expression of this miRNA was suggested as a potential BCR predictor in two studies (Table 4). Correlations were described between the increased expression of miR-21-5p as a BCR predictor and the standard clinicopathological variables (Study nos. 11 and 29; [60,81] while these data were not reported in the controversial studies with the decreased miRNA expression (Study nos. 14 and 31; [64,83]). After adjustment with clinicopathological factors, decreased miRNA expression failed to be an independent BCR risk factor (Study 31, [83]) or was only appropriate in obese patients (Study 14, [64]). Only one of the three studies with upregulated expression in tumor tissue clearly proved miR-21 as an independent factor for shorter BCR-free survival in multivariate analysis (Study 11, [60]).
- **miR-145-5p.** Both a study with increased (Study 5, [52]) and two studies with decreased expression of miR-145 estimated this miRNA as a potential BCR predictor or part of a significant prediction signature (Study nos. 15 and 17; [65,67]). It cannot be excluded that these discrepant findings were caused by analytical reasons, as two studies calculated the expression of miR-145 with normalizers (RNU43 and SNORD48) that were criticized regarding their suitability as reference genes [116]. Another study with decreased miR-145-5p expression (Study 9, [58]) was not able to confirm miR-145-5p as a BCR predictor in Kaplan-Meier analysis. However, it should be noted that the above-mentioned very short follow-up period in that study makes a true assessment difficult.
- **miR-1-3p.** Three studies examined the potential BCR capability of downregulated miR-1. Two studies (Study nos. 8 and 53; [55,107]) identified miR-1 as an independent BCR predictor after adjustment with the conventional clinicopathological factors. However, the additional benefit was not demonstrated when miR-1-3p was included in the model based only on clinicopathological factors. miR-1 was also demonstrated to be a successful BCR predictor in the third study (Study 24, [74]), but its clinical accuracy was exceeded by the pre-operative PSA value. The inconsistent documentation of clinicopathological variables in these studies makes it impossible to attribute this uniform BCR predictor result to congruent clinical characteristics between the studies.
- **miR-96-5p.** In two studies (Study nos. 2 and 17; [23,67]), increased levels of this miRNA in PCa tissue were successfully identified as a single BCR predictor or part of a BCR predictor combination. A third study (Study 9, [58]) did not confirm an association of the recurrence-free survival and the miR-9-5p expression level.

The heterogeneity of results of the particular miRNAs in these multiple studies also reflects the situation of the other miRNAs with only two studies available (Table 4). For example, opposite expression data were reported for miR-133b, but both studies suggested this miRNA as a potential BCR predictor despite their discordant expression data (Study nos. 24 and 26; [74,76]). Moreover, studies that partly use data from publicly available databases or from previous studies may lack clearly defined characteristics complicate objective assessment. This applies to miR-30c-5p (Study nos. 28 and 47; [78,99]) and miR-301-3p (Study nos. 37 and 48; [89,100]).

#### 3.2.3. Multiple miRNAs as Signatures or in Combination with Other Analytes

In the discovery phase of the development of a tissue-based miRNA assay for predicting BCR, highthroughput “-omics” approaches like microarrays or sequencing technologies provide extensive data sets with numerous candidate miRNAs to meet this pursued objective. One approach is to search this pattern of analytes and to select the most effective miRNAs for the validation of BCR prediction in the subsequent development phases. However, there is now a great interest in using this wealth of information not only for selecting single markers but also for combining multiple markers into a specific panel or signature together with clinicopathological data [117,118]. Particular attention should be paid to implement orthogonal markers in such a signature [119]. Orthogonal markers are uncorrelated among each other and to the conventional clinicopathological factors. This uncorrelated particularity is an essential precondition to improve the predictive significance of the signature due to the additional information achieved by these independent factors. For miRNAs, this orthogonal aspect could be demonstrated for the miR-29c-3p, miR-34a-5p, miR-141-3p, and miR-148a-3p that were not associated with tumor size and pathological stage but were inversely correlated with Gleason grades [88]. The Decipher genomic classifier using a 22-gene signature for post-prostatectomy risk stratification or other similar approaches has shown the potential usefulness of such multi-analyte tools [120,121,122].

In this review, the studies by Nam et al. [89] (Study 37: 5-miR signature with miR-139-5p, miR-223-3p, miR-301a-3p, miR-454-3p, and miR-652-3p) and Kristensen et al. [50] (Study 46: 3-miRNA prognostic classifier with miR-185-5p, miR-221-3p, and miR-326) support these ideas. Based on the multiple-miRNA approach as a signature combined with rigorous validation processes (three validations in Study 46) or a high sample size and a high number of BCR events (*n* = 491, 167 BCRs in Study 37), the two studies yielded promising results. Both studies are among the most convincing studies evaluated in this review and can be considered future-oriented examples. Nam et al. [100] focused in a subsequent study (Study 48) on the predictive validity of the single miR-301a-3p from the above-mentioned 5-miRNA signature. The authors also described a good BCR prediction rate using only this single miRNA, but they did not compare the results of the two approaches. Bell et al. [84] published a further BCR prediction study based on a multiple miRNA signature (Study 32). A panel of 88 miRNA was required for a reliable BCR prediction within 3 years after surgery. However, the inclusion of only miR-4516 and miR-601 in a model with Gleason score and lymph node status alone improved the BCR prediction accuracy after salvage radiation treatment from 0.66 to 0.83 of the area under the receiver operating characteristic (ROC) curves. The previously discussed 4-miRNA tool miQ by Larne et al. [67] also proved that the integrated implementation of several differentially regulated miRNAs with orthogonal characteristics improved decision making in the management of PCa patients both in diagnosis and prognosis. Lichner et al. [69] developed three statistical models based on 2 to 3 miRNAs (Study 18: miR-331-3p + miR152-3p, miR-331-3p + miR-152-3p + miR135a-5p, and miR-148a-3p + miR-429) that were verified by internal validation and on an independent cohort. The authors achieved a correct classification rate of 92 to 100% in predicting patients with a high risk of BCR.

The combined use of panels with miRNA and mRNAs is also noteworthy. The mRNAs were either targets of the accompanying miRNAs or independently selected BCR markers, such as those in Study 6 [53] with miR-519, miR-647, and 10 mRNAs, in Study 41 [94] with miR-224-5p and its target APLN or in Study 52 [105] with miR-30d-5p and its target Protein phosphatase 1 regulatory subunit 12A (official symbol: PPP1R12A)(MYPT1).

## 4. Critical Assessment of the Recent Situation of miRNA-Based BCR Prediction

### 4.1. Analytical Considerations

In a previous review on circulating miRNAs in patients suffering from urological tumors, we discussed the typical influential and interfering factors that determine the results of miRNA measurements [51]. These are variables in the collection, further processing and storage of samples in the preanalytical phase, the various isolation and quantification methods based on different principles and technologies in the actual analytical phase and the different normalization strategies in the postanalytical phase. For more details of all these aspects, we refer the interested reader to the overview of Pritchard et al. [123]. In particular, different miRNA extraction procedures for fresh-frozen or FFPE samples and different measurement platforms showed qualitative and quantitative miRNA differences depending on the determination [124,125,126,127]. These differences might especially attribute to the lack of comparability of miRNA profiling data between studies that applied different analytical techniques. On the other hand, this effect needs a strict method harmonization in multi-institutional studies if the analytics are separately performed in every center. New comparative analyses recommended the Qiagen miRNeasy FFPE kit to be the best kit for miRNA isolation from FFPE samples and the new TaqMan advanced miRNA assays as the quantification method of superior sensitivity and specificity in comparison to competitor products [126,128].

All these issues also apply to the evaluated studies in this review and therefore do not need to be discussed again in detail. However, as a concrete example (Table 3, column “Methodology”), it is remarkable that confirmed stably expressed miRNAs for normalizing the expression results were only used in six (12%) of the 53 studies. In contrast, in 33 (63%) of the reviewed studies, different small nuclear and nucleolar RNAs (U6 or RNU6 [official name: RNU6-1], RNU6B [RNU6-6P], RNU43 [SNORD43], RNU44 [SNORD44], RNU47 [SNORD47], RNU48 [SNORD48], and RNU66 [SNORD66]) were used as endogenous normalizers. This was done even though most of these small RNAs were found to be unstably expressed across non-malignant and malignant prostate tissue and therefore considered as less suitable normalizers [116]. The real suitability of RNA47, RNU48, and RNU66 as normalizers was only tested in one study [67]. Thus, the general neglect of analytical basics was obvious in several studies. This was particularly underlined by the fact that none of the reviewed articles referred to the “Minimum Information for Publication of Quantitative Real-Time PCR Experiments” (MIQE) guidelines [129]. These guidelines address the analytical essentials that have to be considered to assess the quality and potential traceability of reverse transcription-quantitative polymerase chain reaction (RT-qPCR) measurements in an extensive checklist. Our observation corresponds with results of a recent survey of over 1700 publications that criticized the frequently insufficient description of experimental details of RT-qPCR measurements in many articles [130]. The authors of that survey called upon journal editors and reviewers to draw more attention to this issue for improving the transparency and comparability of RT-qPCR data between studies. It might be a specific challenge for clinically oriented journals in publishing clinical studies based on modern molecular-biological methods as clinicians often do not place any great emphasis on analytical problems.

### 4.2. Study Design Considerations

Table 3, with the essential details of the evaluated recent miRNA-based BCR studies and our separate comments, illustrates the heterogeneity of the data situation in this field. Different starting points in the discovery phase and specific features in subsequent validation processes hamper a comparison of data between studies. However, to provide a more informative overview not only on the diversity of miRNAs examined but also on the fundamental characteristics between the various studies, we classified various study criteria into categories in Table 5. This facilitates the identification of protocol deficiencies of the particular studies according to the assessment criteria of the development phases for establishing a robust tool in clinical practice (Table 2). Some noteworthy points should be considered more closely in the following.

The different definitions of the PSA cutoff as criterion for the biochemical recurrence were discussed in detail at the beginning of this review. This diversity of cutoffs was also reflected in our survey. Two-thirds of the studies used the cutoff of 0.2 µ/L recommended in the EAU and AUA guidelines [29,30]. However, 23% of the studies did not specify this threshold as a fundamental precondition of data comparability. We also noticed this essential lack of information with regard to the specification of the important risk variables “resection margin status” and “lymph node status” in 60 and 70% of the studies, respectively. In contrast, the pathological tumor stage and Gleason score were generally indicated. On the other hand, only 3% of the studies included PCa patients with PSA values below 10 µg/L. This indicates that few studies focused on low-risk PCa patients.

In addition to these clinicopathological characteristics of the study patients as one part of the study design, more or less formal conditions determine the implementation and, finally, the validity of clinical studies. These study specifications are listed in Table 5 under the category “Study design features”. The percentage data given for the respective items illustrate deficiencies and the limited validity deficiencies of several studies. Thus, studies with sample sizes of less than 50 patients, 10 to 20 BCRs, a mean follow-up period under 5 years, or evaluated only through univariate analysis remain questionable from the statistical and biological point of view. For example, in a multivariate Cox regression analysis as a standard statistical method for BCR analysis, at least ten events per predictor variable are necessary to obtain reliable results [133]. Because several clinicopathological factors have to be individually considered in such a model it is not surprising that a study cohort with 20 BRC events and the additional inclusion of miRNAs of interest can hardly meet a scientifically founded conclusion of clinical significance. Considering a proportion of one-third of patients with BCR as an example, cohorts of more than 150 patients would be advisable. In contrast to this, few (15%) of the studies reviewed here that were exclusively retrospective in nature included more than 150 patients, and those were mostly multi-institutionally implemented. In this regard, it is significant that power and sample size calculations were presented in only two studies (Study nos. 2 and 9; [23,58]). Furthermore, only eight studies (15%) performed an internal or external validation of data that was suggested as an important criterion of the development phase “Validation by clinical assessment” (Table 2; [51]). For example, Kristensen et al. (Study 46; [50]) confirmed the improved prognostic performance of their 3-miRNA prognostic classifier in comparison to the BCR prediction based on only clinicopathological factors in three independent PCa patient cohorts. A similar benefit, proved by increased C-indices, was shown by single miRNAs in two other studies (Study nos. 2 and 30; [23,82]). The proof of such an additional benefit by the inclusion of miRNAs in the conventional model has to be considered as a decisive criterion to proceed further with developing a new clinical decision-making tool. Therefore, it is striking that the authors of merely two studies (Study nos. 2 and 46; [23,50]) pointed out that their studies were performed according to the “Reporting Recommendations for Tumor Marker Prognostic Studies (REMARK)” [131]. The generally “benevolent” neglect of the guideline suggestions in performing the prognostic studies by principal investigators and in accepting final study reports as publications by the journal editors is consistent with the above-mentioned attitude of ignoring the analytical MIQE and “Standards for Reporting of Diagnostic Accuracy (STARD)” guidelines [129,132]. 

### 4.3. Divergences between BCR Outcome and the Functional Role of miRNAs

Divergent BCR outcome data between different studies contrast with the functional data of miRNAs. In addition to the apparent differences due to the previously discussed reasons of the heterogeneity of study results, real divergences seem to exist between the miRNA expression level as a BCR predictor and the potential functional role of the respective miRNA. It is therefore worth briefly mentioning this rarely considered aspect using the examples of let-7c-5p, miR-141-3p, miR-148a-3p, and miR-221.

Leite et al. [52] showed in Study 5 that increased let-7c-5p in the primary untreated PCa tissue was associated with a higher BCR risk. This seems to be in contrast to the generally decreased expression of let-7c-5p in PCa tissue compared with normal prostate tissue and its suppressive action of this miRNA on the androgen receptor [134,135]. However, it should be considered that in the assessment of BCR risk, the expression of let-7c-5p is evaluated only in tumor cells. The BCR indicator effect of the let-7c-5p expression disappeared as an independent factor in the multivariate analysis with all risk factors showing the complex interplay between clinicopathological variables and expression levels of markers [52]. A similar, but contrasting and also not plausibly explainable phenomenon applies to miR-141-3p (Study nos. 31 and 36; [83,88]) and miR-148a-3p (Study 36; [88]). Decreased levels of both miRNAs indicated a shorter recurrence-free survival in the here reviewed studies, whereas their upregulation was found to be increased in untreated PCa and castration-resistant PCa specimens, and these miRNAs enhanced the proliferation of PCa cell lines [134,136,137]. For miR-221-3p and miR-221-5p, decreased expression levels were characteristics of a shorter BCR-free period (Studies nos. 3, 17, 31, 46; [24,50,67,83]. This tumor-suppressive function corresponds with the expression levels and functional data observed in other studies [138,139,140,141]. However, increased expression of miR-221 in PCa metastases and PCa mouse models and an enhanced proliferation of PCa cell lines by this miRNA were also described [142,143,144]. It was recently postulated that this oncogenic role of miR-221 is likely transient, and the dual tumor-suppressive and oncogenic function of miR-221 probably reflects different phases of PCa progression [140]. In this context, the possible divergences between BCR as clinical endpoint and the development-dependent functions of miRNAs would be understandable.

## 5. Future Directions

Despite the discussed critical points and limitations of the reviewed studies, promising results provided by several studies can be considered as proof of the true potential of miRNAs as BCR predictors. It is the final aim of this review to learn from the deficiencies of the conducted studies hitherto and draw corresponding conclusions for future studies. Therefore, our overview of the published results and the background data of the 53 studies allows two essential conclusions:

- No study has thus been able to comply with the suggested requirements specified in the final development phase “Validation of clinical usability” (Table 2) to establish a robust BCR tool for clinical practice using miRNAs. In addition, few studies can be valued as successfully finished in the second development phase due to the lack of internal validation in most of the studies (Table 2 and Table 5).
- The evaluation and comparison of analytical and clinical conditions in the various studies provided a wealth of experience in the assessment of study design features. Based on these experiences, critical study deficiencies could be identified (see Section 4, comments to Table 5), and future directions could be elaborated to overcome these shortcomings. In the following, we focus on some essential issues.

The results of the various studies and their generalized assessment confirm once more the clear need of a good coordination between the intended study aims, all study design elements, and preanalytical and analytical conditions. The three guidelines MIQE, REMARK, and STARD should be strictly considered in future studies since they define the basic foundation for implementing a study under common clinical and analytical conditions [129,131,132]. For planned projects, especially prospective, multi-institutional studies, appropriate elements of these guidelines should be specified, and their compliance should be a subject of constant control to guarantee necessary preconditions for a reliable database. These guidelines not only allow the necessary transparency but also the harmonization and comparability of results between multi-institutional studies. However, because of numerous factors, such as different methods of RNA isolation, reverse transcription, and true miRNA measurements, as well as various platform applications that could influence RT-qPCR results, it is advisable to perform all analyses at one institution in early studies. This approach would a priori avoid misinterpretations, as errors can be excluded due to missing traceability between results obtained through different methods. The issue of analytical differences could be solved later in a second step of method harmonization. The same applies to retrospective studies. 

While BCR does not equal clinical relapse, elevated postoperative serum PSA levels make it possible to filter patients with a high risk of true disease recurrence [41]. Therefore, future studies should additionally focus on the predictive capability of miRNAs with regard to the clinical endpoints of distant metastasis, cancer-specific death, and response rate to drugs. Some studies (Study nos. 3, 14, 32, 53; [24,64,84,107]) have already considered these endpoints. However, the “mixed” use of these endpoints should be avoided in future studies because the distinct time difference between the endpoints could result in a systematic bias. In this respect, the study design should also clearly address the specific need for different patient groups. For example, the predictive tools using miRNAs differ between low-risk and high-risk patients following radical prostatectomy [69,71]. This result, which is also shown by using other genomic classifiers [122], should be considered in an adapted composition of the study groups according to the specific clinical objective. In addition, the Gleason-related association of miRNAs shown exemplarily by Lichner et al. [88] requires a re-assessment according to the new International Society of Urological Pathology (ISUP) Gleason group classification. Further multi-institutional studies are needed to validate the clinical usability of miRNA-based tools, either alone or combined with clinicopathological factors, for BCR prediction. The additional information provided by miRNAs in comparison to established BCR prediction tools [4,5,6] must be proven in these studies and should be demonstrated by decision curve analysis [145]. It is worth considering whether the above described 2–5 miRNA signatures could be confirmed in comparison to these clinically established tools in retrospective multi-institutional approaches as a validation step to initiate prospective studies.

## 6. Conclusions

In summary, miRNAs were shown in several studies of this review as promising marker candidates and miRNA signatures for predicting BCR after radical prostatectomy. However, the general non-consideration of the MIQE, REMARK, and STARD guidelines in most studies resulted in study design deficiencies, primarily a lack of internal validation of data. The unequivocal evidence of additional information through miRNAs in comparison to the conventional approaches of BCR has not been proven thus far. Further studies are needed to address these deficiencies both in retrospective and prospective multi-institutional studies to validate the clinical usability and benefit of miRNA-based BCR tools in combination with the conventional clinicopathological variables.

## Figures and Tables

**Table 1:** Factors influencing the “biochemical recurrence” diagnosis after radical prostatectomy.

Factors	Comments	References
1. Definition of biochemical recurrence	Use of different PSA cutoffs combined with or without other criteria for estimation of biochemical recurrence	[27,31,37,42,43,44]
2. Assay-dependent PSA concentrations	Lack of metrological traceability between different PSA assays because of biological (PSA heterogeneity) and methodological reasons (use of different antibodies with different epitope specificities and affinities; different technical principles)	[45,46]
3. Clinicopathological particularities	Age and ethnic disparities; adverse tumor characteristics (TNM classification, Gleason score or ISUP grade groups; risk classification of patients); surgical complications (positive margins)	[5,6,32,33,34,47,48]
4. Duration of follow-up	The selected follow-up period after surgery decisively determines the total number of observed events of biochemical recurrence	[41,47]

PSA, prostate-specific antigen; TNM, classification of malignant tumors describing the involment of the primary tumor, regional lymph nodes, and the distant metastatic spread; ISUP, International Society of Urological Pathology.

**Table 2:** Development phases to use miRNAs as predictors of biochemical recurrence after radical prostatectomy.

**1.** **Discovery and selection of potential miRNAs**
Identification and selection of differentially expressed miRNAs based on various principles miRNA-wide profiling in prostatectomy tissue samples on array/sequencing basis Selected differentially expressed miRNAs in prostatectomy tissue samples from recurrent and non-recurrent patients Selected specific miRNAs from prostate cancer cell lines Selected miRNAs based on bioinformatic analyses and pathway data
**2.** **Validation by clinical assessment**
Proof as BCR predictor in retrospective/mono- or multi-institutional studies with internal validation
**3.** **Validation by clinical usability**
Proof in prospective, multi-institutional studies as advantageous or non-inferior tool in comparison to standard procedures in the decision making process of the clinical management of patients

Adapted from Fendler et al. [51]. BCR, biochemical recurrence.

**Table 3:** Studies regarding tissue miRNAs as predictive markers for biochemical recurrence in prostate cancer after radical prostatectomy.

No.	Reference, year	Study Details in the Marker Development Phases ^1^	Sample	Methodology ^2^	Significant miRNAs ^3^	Statistical Methods and Results	Assessment of the Presented Clinical Findings
1	Tong et al., 2009 [22]	Discovery: 20 early BCR pat. (<2 years after RP) vs. 20 non-BCR pat. (>10 years after RP). Validation: 11 early BCR vs. 11 non-BCR. BCR: PSA criterion not defined.	FFPE	Discovery: microarray, Validation: RT-qPCR (TaqMan) by analysis of 6 miRs; RM: synthetic RNA.	miR-135b-5p ↑ miR-194-5p ↑	Ratio of BCR to non-BCR: 1.6 for miR-135b and 1.4 for miR-194, but *p* > 0.050) with MW-test.	Aberrant expression of miR-135b and miR-194 may only reflect a tendency for early disease relapse. Low sample size.
2	Schaefer et al., 2010 [23]	Discovery: 24 matched normal and malignant tissue samples and literature data. Validation with two independent cohorts: 1) 76 pat., median follow-up of 50 months after RP, 12 BCR. 2) 79 pat., median follow-up of 50 months, 14 BCRs. BCR: PSA >0.1 ng/mL, confirmed by at least one subsequent increasing value.	Fresh-frozen tissue	Discovery: Agilent microarray. Validation: RT-qPCR (TaqMan) by analysis of 15 dysregulated miRs; RM: miR-130b-3p.	miR-96-5p ↑	(1). KMA of RFS: log-rank test, *p* = 0.039. (2). CoxM: HR = 3.20, *p* = 0.023, independent factor for BCR in the combined cohorts.	Increased miR-96 can be considered as a BCR predictor in combination with the Gleason score.
3	Spahn et al., 2010 [24]	Discovery: 4 pairs of primary carcinoma and metastasis tissues vs. 4 BPH tissues. Validation of clinical utility: 92 high-risk patients with PSA >20 µg/L and positive lymph node status in >50% median follow-up of 74 months. BCR: PSA ≥0.2 ng/mL on 2 consecutive follow-up visits.	FFPE	Discovery: in-house microarray analysis Validation: RT-qPCR (TaqMan) by analysis of 4 out of 14 dysregulated miRs in a limited sample size and later of miR-221 in the high-risk cohort; RM: RNU6B.	miR-221-3p ↓	(1). KMA of RFS: log-rank test, *p* < 0.01. (2). CoxM: HR = 0.525, *p* = 0.032, combined with Gleason score and tumor stage, calculated relative to clinical recurrence (local or distant metastatic disease) but not BCR.	miR-221 downregulation was linked to clinical recurrence in a high-risk PCa cohort as independent factor.
4	Fendler et al., 2011 [25]	Discovery: 10 BCR pat. (<1 year after RP) vs. 10 BCR pat. (>1–4 years) vs. 10 non-BCR pat. (within 3 years). Validation: 24 BCR pat. (<1 year) vs. 22 non-BCR pat. (within 2 years). BCR: PSA >0.1 ng/mL confirmed by at least one subsequent increasing value.	FFPE	Discovery: TaqMan array. Validation: RT-qPCR (TaqMan) of out of 65 dysregulated miRs; RM: RNU44.	miR-10b-5p ↑	(1). KMA of RF of only miR-10b: log-rank test, *p* = 0.023). (2). ROC of RFS: AUC = 0.72. (3). CoxM: HR = 2.10, *p* = 0.033.	miR-10b remained the only predictor variable of BCR in a multivariate Cox regression model.
5	Leite et al., 2011 [52]	Discovery: 14 selected miRNAs based on miR-based prediction of Target genes (TargetScan). Validation: 21 BCR vs. 28 non-BCR, follow-up <10 years. BCR: postoperative PSA ≥0.2 µg/L.	Fresh-frozen tissue	14 miRs were analyzed by RT-qPCR (TaqMan); RNU43.	miR-100-5p ↑ miR-145-5p ↑ miR-191-5p ↑ let-7c-5p ↑	(1). KMA of RFS for the 4 miRs: log rank test, *p* < 0.05. (2). CoxU for the 4 miRs: HRs at least with *p* < 0.05. (3). CoxM: miR-100 (HR: 3.68, *p* = 0.009), independent factor in addition with tumor volume.	High levels of miR-100, miR-145, miR-191, and let-7c were related to BCR; miR-100 with highest impact in multivariate model.
6	Long et al., 2011 [53]	Discovery: 29 BCR pat. median 19 months after RP) vs. 41 non-BCR pat. (median 83 months). Validation: independent cohort (13 BCR pat. vs. 27 non-BCR pat. BCR: two detectable PSA >0.2 ng/mL.	FFPE	Integrated DASL assays (Illumina) for mRNAs and miRNAs; RM: quantile normalization.	10 mRNAs miR-647 ↓ miR-519 ↑	Use of the combined mRNA-miRNA panel; KMA and CoxM: at least *p* < 0.05 of BCR prediction in discovery and validation sets.	Prediction model of the mRNA-miRNA combined with clinicopathological data outperformed the model based on only clinicopathological data.
7	Barron et al., 2012 [54]	18 PCa pat. after RP with BCR (<2 years) matched with 18 pat. without BCR (>3 years) according to pT3, similar Gleason score, and preoperative PSA. BCR: PSA criterion not defined.	FFPE	RT-qPCR (TaqMan); RM: RNU48.	miR-200a-3p ↓	Student’s *t*-test *p* = 0.057	Unclear BCR prediction evidence of miR-200a underexpression although miR-200a overexpression reduced PCa cell growth.
8	Hudson et al., 2012 [55]	Discovery: miR-1 and miR-133a were selected based on a previous study [56]. Validation: 99 PCa samples and data from another study [57], unclear consideration of clinical factors and number of BCRs. BCR: postoperative PSA ≥0.2 µg/L on two occasions.	Fresh-frozen tissue	RT-qPCR (TaqMan); RM: U6.	miR-1-3p ↓	(1). KMA for RFS: log-rank test, *p* = 0.008. (2). CoxM: HR = 0.29 of high vs. low miR-1 in a model adjusted with clinicopathological factors.	Reduced miR-1 was considered a potential BCR risk factor.
9	Kang et al., 2012 [58]	Intention to confirm miR-96, miR-145, and miR-221 as potential BCR predictors as shown in previous studies [22,23,24]. Validation: 73 PCa pat., 14 BCRs, mean follow-up of 19.4 months. BCR: PSA ≥0.2 µg/L at 2 consecutive follow-up visits.	FFPE	RT-qPCR (TaqMan); RM: RNU6.	miR-96-5p-5p (-) miR-145-5p (-) miR- 221-3p (-)	KMA, CoxU and CoxM: no significant BCR prediction with all three miRs.	None of the 3 miRs could be confirmed as BCR predictors; however, the follow-up period was <2 years.
10	Kobayashi et al., 2012 [59]	Discovery: Unfounded selection of miR-30d as one of 3 miRs with a >2-fold increased expression in PCa cell lines. Validation: 56 PCa pat. after RP with 10 BCR events. BCR: continuously elevated PSA >0.2 µg/L.	Fresh-frozen tissue	Discovery: microarray (Toray, Japan). Validation: RT-qPCR (TaqMan); RM: RNU6B.	miR-30d-5p ↑	(1). No association with all standard clinicopathological factors but with BCR. (2). CoxM: in a model adjusted with all standard clinicopathological factors only the combination of high miR-30d and reduced level of its target SOCS remained as the only significant BCR predictor (HR: 4.447, *p* = 0.004).	miR-30d-overexpression and low SOCS expression seems to be a relevant orthogonal marker combination of early BCR prediction.
11	Li et al., 2012 [60]	Discovery: miR-21 was found an oncogenic miR in a previous cell line study [61]. Validation: 116 BCR pat. vs. 52 non-BCR pat., with 78 low and 90 high miR-21 expressions. BCR: postoperative PSA of ≥0.2 µg/L.	FFPE	Immuno-reactivity of miR-21 by locked nucleic acid in situ hybridization (Exiqon); RM: not defined.	miR-21-5p ↑	(1). KMA: increased miR-21 with shorter RFS, log rank test, *p* = 0.001. (2). CoxM: HR: 2.059, *p* = 0.029 as independent BCR predictor together with PSA in a model adjusted with standard clinicopathological factors.	High miR-21 expression was associated with poor BCR-free survival and can predict the risk of BCR.
12	Majid et al., 2012 [62]	Discovery: downregulated miR-23b were found in PCa cell lines. Validation: 151 PCa tissues samples to confirm low expression of miR-23b in malignant vs. non-malignant tissue samples; 105 samples used for BCR prediction, number of BCR not given. BCR: PSA criterion not defined.	Fresh-frozen tissue	Discovery: microarray of cell lines. Validation: RT-qPCR (TaqMan); RM: U6.	miR-23b-3p ↓	(1). KMA of RFS: log-rank test *p* < 0.002. (2). Multiple regression analysis (but not CoxM) showed miR-23b as an independent BCR predictor (*p* < 0.02).	Low miR-23b expression was obviously associated with a short RFS; however, corresponding multivariate Cox regression analyses were not performed.
13	Saini et al., 2012 [63]	Differential expression of paired malignant to non-malignant miR-708 expression in 22 BCR pat. vs. 70 non-BCR pat. BCR: PSA level not defined.	FFPE	RT-qPCR; RM: RNU48.	miR-708-5p ↓	Only the statement that 18 of the 22 BCR pat. had reduced miR-708 expression.	Clinical evidence of low miR-708 expression as BCR predictor was not statistically presented.
14	Amank-wah et al., 2013 [64]	Selection of miR-21, miR-221, and miR-222 as potential predictors of BCR based on literature data and the possible relationship between obesity and recurrence. Validation: 28 recurrent vs. 37 non-recurrent PCa. Recurrence criterion in this study: postoperative PSA ≥0.2 µg/L or clinical metastasis or cancer specific death.	FFPE	RT-qPCR (TaqMan); RM: RNU6B.	miR-21-5p ↓ miR-221-3p (-) miR-222-3p (-)	(1). KMA of RFS: significant log rank test only for miR-21, *p* = 0.0001. (2). CoxM: low miR-21 in age-adjusted model predicted recurrence in obese (HR: 5.40, *p* = 0.031), but not in non-obese patients.	miR-21 was only associated with PCa recurrence in obese patients, but no evidence was provided in multivariate models with all standard clinicopathological variables.
15	Avgeris et al., 2013 [65]	Intention to confirm decreased miR-145 as potential BCR predictor as shown in previous studies. Validation: 62 PCa pat. with follow-ups >40 months, 32 BCRs. BCR: 2 consecutive measurements of PSA ≥0.2 µg/L.	Fresh-frozen tissue	RT-qPCR (SYBR-Green); RM: SNORD48.	miR-145-5p ↓	(1). KMA for RFS: log-rank test *p* = 0.027. (2). CoxM: low miR-145 remained as the only significant unfavorable BCR predictor (HR: 4.467, *p* < 0.02).	Low miR-145 expression outperformed the BCR prediction through standard clinicopathological factors.
16	He et al., 2013 [66]	Discovery: 4 pairs of primary PCa and adjacent benign tissue. Validation: 104 PCa pat. with 27 BCRs but follow-up time not indicated. BCR: PSA level not defined.	Fresh-frozen tissue	Discovery: Microarray (Agilent). Validation: RT-qPCR (GeneCopoeia) and MIRCURY hybridization (Exiqon); RM: RNU6B and miR-130b-3p.	miR-374b-5p ↓	(1). KMA for RFS: log-rank test, *p* = 0.005. (2). CoxM: miR-374b (HR = 0.38, *p* = 0.018) remained as an independent BCR predictor together with the Gleason score.	Low miR-374b was identified as an independent BCR predictor, specifically in Chinese patients.
17	Larne et al., 2013 [67]	Discovery: based on microarry data of Martens-Uzunova et al. [68] of 50 primary PCa and 11 normal adjacent tissue samples, BCR events not given. Validation for BCR: 52 PCa pat. of cohort 2, number of BCRs not indicated. BCR: consecutive PSA levels >0.2 µg/L or one single >1 µg/L.	FFPE	Discovery: Microarray (Agilent). Validation: RT-qPCR (Exiqon). RM: geometric mean of RNU47, RNU48, RNU66.	miR-96-5p ↑ miR-145-5p ↓ miR-183-5p ↑ miR-221-5p ↓	Ratio of (miR-96 x miR-183/miR145 x miR-221) was constructed to discriminate between malignant and non-malignant prostate tissue but also predict aggressiveness, metastasis, overall survival, and BCR risk; internal and external validation was performed.	This ratio termed as miQ (miRNA index quote) might be very useful as indicated; however, its use for BCR prediction remains unclear despite the significant KMA, as the relationship and benefit to other clinicopathological variables were not shown.
18	Lichner et al., 2013 [69]	Discovery: 27 BCR pat. (<3 years) vs. 14 non-BCR pat. (>3 years). Validation: independent cohorts with 35 and 29 corresponding patients. BCR: PSA criterion not defined.	FFPE	Discovery: TaqMan array card A + B. Validation: RT-qPCR (TaqMan); RM: RNU48.	miR-152-3p ↓ miR-331-3p ↑	(1). Differential expression of 25 miRs between the 2 BCR groups; 16 miRs significantly discriminated (ROC analysis) between them. (2). Three developed logistic regression models with 2–3 miRs correctly classified with >90%.	miR-331-3p and miR-152 were most useful both in the discovery and validation set and could predict BCR risk at the time of prostatectomy.
19	Majid et al., 2013 [70]	Intention: to validate miR-34b expression as a BCR prediction tool and identify its functional role. Validation: 74 pairs of matched tissue samples, 17 BCRs, follow-up period not given. BCR: first postoperative PSA >0.1 µg/L) after at least one undetectable PSA (<0.04 µg/) after RP.	Fresh-frozen tissue	RT-qPCR (TaqMan); RM: not defined.	miR-34b-3p ↓	KMA: low expression was associated with shorter RFS (log-rank test, *p* = 0.02).	Low miR-34b might have prognostic value in BCR prediction but that was not assessed by multivariate analysis.
20	Schubert et al., 2013 [71]	Discovery: 13 high-risk PCa cases and 6 BPH. Validation: 2 independent, two-centric cohorts of 98 and 92 high-risk PCa pat., mean follow-ups >6.5 years but BCR events not reported. BCR: PSA ≥0.2 µg/L on 2 consecutive follow-up visits.	FFPE	Discovery: microarray analysis. Validation: RT-qPCR (TaqMan); RM: RNU6B.	let-7b-5p ↓	Specific miR signatures of high-risk PCa patients with different clinical outcomes were identified. CoxM: let-7b was validated in the 2 validation cohorts as independent BCR predictor (HR: 0.44 and 0.30, *p* ≤ 0.05) together with the Gleason score.	Low let-7b expression was successfully validated as a predictor of BCR and clinical failure (local or distant metastasis) in high-risk PCa patients.
21	Sun et al., 2013 [72]	Intention to examine the clinical significance of miR-126 as it is known as a regulator in other tumors. Validation: 128 PCa tissue samples, follow-up from 3 to 10 years, BCRs not indicated. BCR: PSA ≥0.2 µg/L on 2 consecutive follow-up visits.	Fresh-frozen tissue	RT-qPCR (TaqMan); RM: RNU6B.	miR-126-3p ↓	(1). KMA of RFS: log-rank test, *p* < 0.001. (2). CoxM: low miR (HR = 3.68, *p* = 0.01).	miR-126 expression, tumor stage and lymph node status were identified as independent BCR predictors.
22	Avgeris et al., 2014 [73]	Discovery: Based on the reduced miR-378 expression in PCa tissue [68], the regulatory role of this miR on kallikrein 2 and 4 as PCa elements was predicted in silico. Validation: 62 PCa tissue samples, median follow-up <5 years with 32 BCRs. BCR: PSA ≥0.2 µg/L by 2 consecutive measurements.	Fresh-frozen tissue	RT-qPCR; RM: SNORD48.	miR-378a-3p ↓	(1). KMA of RFS: reduced miR-378 discriminated Gleason 3 + 4 and 4 + 3 in patients with worse RFS (log-rank test, *p* < 0.001). (2). CoxM: only in high and very-high-risk PCa pat. was the loss of miR-378 an independent BCR predictor together with the Gleason score but not in the whole cohort.	Loss of miR-378 expression showed a limited capability of BCR prediction only in high-risk PCa pat.
23	Casanova-Salas et al., 2014 [49]	Discovery: differential miR expression in 50 PCa tissue vs. 10 normal tissue samples. Validation: analytical validation in the discovery set, clinical validation in independent samples from 122 BCR vs. 151 non-BCR pat., mean follow-up time 7.7 years. BCR: PSA ≥0.4 µg/L during follow-up.	Fresh frozen tissue; FFPE	Discovery: microarray (Applied) Validation: RT-qPCR (TaqMan); RM: RNU44 and RNU48.	miR-182-5p ↑ miR-187-3p ↓	(1). miR-182/-87 as the most dysregulated miRs were further analyzed. (2). KMA: high miR-182 predicted shorter RFS, also within the Gleason score groups. (3). CoxM: miR-182 was an independent factor, combined with the Gleason score especially for Gleason score 7.	miR-182 in combination with the Gleason score showed a promising capability for BCR prediction but not for clinical progression.
24	Karatas et al., 2014 [74]	Discovery: 20 BCR vs. 20 non-BCR pat. Validation: independent 21 BCR vs. 21 non-BCR pat., mean follow-up <5 years. BCR: PSA ≥0.2 µg/L by 2 on 2 consecutive follow-up visits.	Fresh-frozen tissue	Discovery: microarray (Agilent). Validation: RT-qPCR (TaqMan) of selected miRs; RM: RNU43.	miR-1-3p ↓ miR-133b ↓	(1). Reduced expression of both miRs in BCR samples (Student’s t-test, *p* < 0.05. (2). ROC analysis: miR-1 with AUC 0.661; miR-133b with AUC 0.692, but PSA 0.950.	miR-1 and miR-133b predicted between BCR and non-BCR pat.; however, PSA clearly outperformed their BCR prediction. Multivariate analysis was missing.
25	Katz et al., 2014 [75]	Discovery: identification of miRNAs as potential modulators of epithelial-mesenchymal transition based on literature search. Validation: 51 PCa pat., mean follow-up 5.3 years with 17 BCRs. BCR: PSA ≥0.02 µg/L.	Fresh-frozen tissue	RT-qPCR (TaqMan); RM: RNU48.	miR-200b-3p ↓	KMA of RFS: low miR-200b resultet in shorter RFS (log rank test, *p* = 0.049). Multivariate analysis was not performed.	Functional significance of miR-200b for epithelial-mesenchymal transition verified but not for BCR compared with standard clinicopathological factors.
26	Li et al., 2014 [76]	Intention to identify the role of miR-133b as a tumor suppressor as shown in other cancers. Validation: 135 PCa tissue samples, follow-up <5 years with 71 BCRs. BCR: postoperative PSA ≥0.2 µg/L on 2 consecutive follow-up visits.	Fresh-frozen tissue	MIRCURY hybridization (Exiqon); RM: not defined.	miR-133b ↑	(1). KMA of RFS: log-rank test, *p* = 0.032. (2). CoxM: HR = 1.775, *p* = 0.045.	Increased miR-133b expression, Gleason score, pre-operative PSA, and tumor margin status were identified as independent BCR predictors. Downregulated RB1CC1 protein as target of miR-133b acted as poor BCR predictor accordingly.
27	Lin et al., 2014 [77]	Discovery: Based on a previous microarray study [66] and studies in other tumors, miR-224 was identified as potential candidate. Validation: 114 PCa samples, follow-up from 0.2 to 14 years, BCRs not indicated. BCR: PSA ≥0.2 µg/L on two occasions.	FFPE	RT-qPCR (GeneCopoeia) and MIRCURY hybridization (Exiqon); RM: RNU6B.	miR-224-5p ↓	(1). KMA of RFS: low expression with shorter RFS, log rank test, *p* = 0.017. (2). CoxM: HR = 0.25, *p* = 0.010.	Reduced miR-224 expression, tumor stage and the Gleason score were identified as independent BCR predictors. Upregulated TRIB1 protein as target of miR-224 corresponded as poor BCR predictor.
28	Ling et al., 2014 [78]	Discovery: Based on previous studies [79,80] that miR-30c acts as potential candidate. Validation: 103 pairs of tumor tissues and adjacent benign tissues, median 3.7 years after RP with 25 BCRs. BCR: postoperative PSA ≥0.2 µg/L.	Fresh-frozen tissue	RT-qPCR (GeneCopoeia); RM: RNU6B.	miR-30c-5p ↓	(1). KMA: low expression with shorter RFS, log rank test, *p* = 0.023. (2). CoxM: HR = 0.34, *p* = 0.002.	Reduced miR-30c expression, tumor stage and the Gleason score were identified as independent BCR predictors.
29	Melbø-Jørgensen et al., 2014 [81]	Discovery: 14 PCa pat. with BCR within 24 months vs. 16 non-BCR. Validation: 535 PCa tissue samples, median follow-up 7.4 years with 170 BCRs. BCR: PSA ≥0.4 µg/L.	FFPE	Discovery: microarray. Validation: RT-qPCR, in situ hybridization (Exiqon); RM: miR-23b-3p.	4 up- and 3 downregulated miRs in the discovery step. Only miR-21-5p↑ was significantly validated.	(1). Higher miR-21 expression in tumor stroma than in tumor epithelial cells. (2). KMA of shorter RFS: log rank tests of high miR-21 in tumor stroma and Gleason score 6, *p* = 0.006 and *p* = 0.023. (3). CoxM for BCR: HR = 2.40, *p* = 0.037 for high stromal miR-21 in patients with Gleason 6, but only *p* = 0.08 for total cohort.	Upregulation of miR-21 was associated with BCR only in tumor stroma and only in low risk patients. Detection needs a more complicated and less convenient method than the in situ hybridization method.
30	Mortensen et al., 2014 [82]	Discovery: 22 BCR vs. 14 non-BCR pat. Validation: Independent 163 PCa cases, median follow up 5.5 years, 96 BCRs. BCR: postoperative PSA >0.2 µg/L on 2 consecutive follow-up visits.	FFPE	Discovery: TaqMan card A + B analysis, miR-449b ↑: 2.8 times higher in BCR than in non-BCR compared to other 31 dysregulated miRs. Validation: RT-qPCR (TaqMan); RM: MammU6.	miR-449b-5p ↑	(1). KMA of RFS: log rank test, *p* = 0.026. (2). CoxM: HR = 1.90, *p* = 0.003. 3. Overall prediction accuracy: Harrell’s C index combined with clinical factors was 0.71.	High miR-449b expression was combined with tumor stage, Gleason score, preoperative PSA an independent BCR predictor.
31	Zheng et al., 2014 [83]	Discovery: Previous studies found dysregulated miR-21, miR-141, and miR-221 in PCa tissue. Validation: 59 BCR vs. matched paired 59 non-BCR pat. Recurrence: BCR with postoperative PSA >0.2 µg/L or local or distant metastasis or cancer-specific death.	FFPE	RT-qPCR (TaqMan); RM: RNU6.	miR-21-5p ↓ miR-141-3p ↓ miR-221-3p ↓	(1). Wilcoxon test with reduced miR levels in BCR vs. non-BCR pat., *p* < 0.02 for the 3 miRs. (2). CoxM: only miR-221 remained as an independent BCR predictor after multivariable adjustment.	Localized PCa pat. with lower miR-221 expression may have a greater risk for cancer recurrence after surgery.
32	Bell et al., 2015 [84]	43 PCa pat. after RP and salvage radiation therapy radiation therapy, 19 with early BCR after RP <3 years and 24 with late BCR >3 years, median follow-up of 6.9 years. Recurrence: BCR as PSA ≥0.2 µg/L on 2 consecutive follow-up visits and clinical recurrence as local, regional and systemic recurrence.	FFPE	Nanostring microarray with 800 miRNA probes; RM: geometric mean approach.	Different miRNA signatures for different objectives. miR-4516 ↑ miR-601 ↑	(1). CoxM for first BCR after RP: 88 miRNA signature combined with D'Amico and Stephenson scores; all single miRs and in combination with significant HRs. (2). CoxM for first BCR after salvage radiation: significant 9 miRNA signature. (3). miR-4516 and miR-601 combined with the Gleason score and lymph node status significantly improved the prediction of BCR after salvage radiation compared to only clinical factors (AUC of 0.83 vs. 0.66).	The developed models with the 88-miRNA signature and the two-miRNA signatures (miR-4516 and miR-601) combined with clinicopathological factors underline the impact of miRNAs to improve the predictive BCR capability of tools based on only clinicopathological factors. Valuable additional bioinformatic data.
33	Cai et al., 2015 [85]	Discovery: miR-195 was selected as a potential BCR marker according to the Taylor data set. Validation: use of the data of Taylor et al. [57], 61 BCR pat. vs. 137 non-BCR pat. with mean follow-up 4 years. BCR: PSA ≥0.2 µg/L on two occasions according to Taylor et al. [57].	FFPE	Microarray	miR-195-5p ↓	(1). MW test: lower level miR-195 in BCR vs. non-BCR pat. (*p* < 0.05). (2). KMA: shorter RFS in low miR-195 vs. high miR-195, *p* = 0.022. 3. CoxM: miR-195 and the Gleason score remained independent BCR predictors.	Decreased expression of miR-195 predicted BCR.
34	Guo et al., 2015 [86]	Discovery/background: miR-195 was examined based on re-analysis of the Taylor data set [57] with decreased miR-195 in PCa tissue. Validation: 31 BCR vs. 109 non-BCR pat., follow-up time not given. BCR: PSA criterion not indicated.	Fresh-frozen tissue	RT-qPCR (TaqMan); RM: RNU6.	miR-195-5p ↓	(1). Association of low miR-195 expression with recurrence (Chi-square, 0.002). (2). CoxU, -M: HR = 5.98 and 5.96, *p* < 0.001 and 0.031. miR-195, the Gleason score and lymph node status remained as independent factors in the multivariate model.	miR-195 improved the BCR prediction in a model combined with conventional clinicopathological factors.
35	Leite et al., 2015 [87]	Discovery: 13 BCR vs. 40 non-BCR pat. Validation: 51 of the discovery group and additional 37 BCR and 39 non-BCR pat. with follow-ups up to 10 years. BCR: postoperative PSA >0.2 µg/L.	Fresh-frozen tissue	Discovery: microarray (Affymetrix). Validation: RT-qPCR (TaqMan); RM: RNU43.	miR-21-3p ↑ of the 31 dysregulated miRs identified in discovery were further validated.	(1). Student’s t-test: mean expression in BCR group 7.20 vs. 2.21 in non-BCR group, *p* = 0.014. (2). KMA: high miR-21 resulted in shorter BCR-free survival (*p* = 0.003). (3). CoxM: HR = 2.5 for miR-21 was the sole independent BCR predictor in a model with all standard clinicopathological factors.	High level of miR-21 seems to be associated with BCR. However, detailed data of the multivariate model were not shown.
36	Lichner et al., 2015 [88]	Discovery: 45 PCa patients, 15 of each with a Gleason grade of 3, 4 or 5. Validation 1: independent 60 PCa after RP to validate relationship between miRNAs and Gleason grade. Validation 2: 23 high risk BCR pat. (≤2 years) vs. 37 low risk BCR pat. BCR: PSA criterion not indicated.	FFPE	Discovery: TaqMan miRNA array cards A + B. Validation: RT-qPCR (TaqMan); RNU6, RNU44 and RNU48.	miR-29c-3p ↓ miR-141-3p ↓ miR-148a-3p ↓ miR-34a-5p ↓	(1). Indicated miRs showed a decreased expression with increasing Gleason grade. (2). MW test: high-risk vs. low-risk BCR pat. for miR-29c, miR-141, miR-148a, *p* < 0.05 and also BCR vs. non-BCR regardless of the time of BCR.	Identification of Gleason grade-dependent of miRNAs that were related to BCR. Not evaluated by multivariate analysis. Detailed bioinformatic information based on experimental work.
37	Nam et. al 2015 [89]	Discovery: 18 PCa pat. with metastasis and 13 non-BCR within 5 years after RP. Validation: 491 PCa patients (167 with BCR and 25 with metastasis), median follow-up 8.7 years. BCR: PSA >0.2 µg/L on 2 consecutive follow-up visits.	FFPE	Discovery: Next-generation miRNA sequencing. Validation: RT-qPCR (Qiagen); RM: miR-28-5p.	Out of 33 potential candidates, 5 miRs were selected for validation: miR-301a-3p ↑ + miR-652-3p ↑ + miR-454-3p ↑ + miR-223-3p ↓ + miR-139-5p ↓	(1). This 5-miR panel predicted metastasis with ROC-AUC of 95.3% in the discovery set. (2). CoxU,-M: HR = 3.9 and 2.6, always *p* = 0.0001 for this miR panel in the validation set. The miR panel remained an independent factor in the multivariate model together with the Gleason score, tumor stage, and PSA.	This 5-miR signature could be used as a potential new and promising prognostic factor combined with known clinicopathological factors to improve the clinical management of patients after RP. Until now, it is one of the most convincing studies.
38	Sun et al., 2015 [90]	Discovery: previous study [91] on regulatory role of miR-128 in PCa cell invasion resulted in the aim of this study with a focus on the prognostic role of miR-128. Validation: 128 PCa pat., follow-up after RP between 3 and 10 years, number of BCRs not given. BCR: PSA ≥0.2 µg/L on 2 consecutive follow-up visits.	Fresh-frozen tissue	RT-qPCR; RM: RNU6B.	miR-128-3p ↓	(1). KMA and CoxU: low level of miR-128 predicted a shorter RFS, log rank test, *p* < 0.001. (2). CoxM: HR = 3.96, *p* < 0.01, remained with tumor stage and lymph node status as independent factors in the model.	Decreased expression of miR-128 was proved to be an independent predictor of the BCR-free survival.
39	Tian et al., 2015 [92]	Based on the significance of stem cells in cancerogenesis, 6 miRs previously reported as differentially expressed miRs in PCa stem cells were tested as BCR predictors.: 32 BCR (within <4 years) vs. 36 non-BCR (≥4 years) pat. BCR: PSA >0.2 µg/L on 2 consecutive follow-up visits.	Fresh-frozen tissue	RT-qPCR (TaqMan); RNU43.	let-7a-5p ↓	Only let7a was significantly downregulated in BCR pat. No further statistical evaluation in combination with clinicopathological variables.	Let-7a may be functionally involved in PCa cancerogenesis; however, its role as a BCR predictor remains an unsolved question in this study.
40	Wallis et al., 2015 [93]	Discovery: based on a previous Study, i.e., 22 [49], miR-182 was examined to obtain more information on the functional role of this miR. Validation: intended as external validation of [49] with 50 BCR and 50 non-BCR pat., median follow-up 5 years. BCR: PSA increase of ≥0.2 µg/L on at least 2 consecutive follow-up visits.	Fresh-frozen tissue	RT-qPCR (Qiagen); RNU6B.	miR-182-5p (-)	miR-182 was not associated with BCR according to the interpretation of the data by the authors; the used statistical methods (univariate and multivariate logistic regression) did not consider the follow-up time frame.	This study should not be considered as external validation of Study 22 [49].
41	Wan et al., 2015 [94]	Discovery: based on previous studies of the authors [77,79] with decreased miR-224 as potential modulator of its target apelin. Validation: 20 matched pairs of PCa for miR-224/apelin axis and 104 PCa data of the Taylor data set [57]. BCR: PSA threshold not reported, probably postoperative PSA ≥0.2 µg/L on two occasions according to Taylor et al. [57].	Fresh-frozen tissue	Discovery: microarray. Validation: microarray and RT-qPCR (GeneCopoeia); RM: RNU6B.	miR-224-5p ↓, combined with its increased target APLN mRNA	(1). KMA of RFS: low miR-224 + high APLN vs. high miR-224 + low APLN 224 with shorter BCR-free survival, log-rank test, *p* = 0.031. (2). CoxM: miR-224 and APLN mRNA could not be confirmed as independent BCR predictors (*p* > 0.3).	The association of the dysregulated miR-224/APLN axis to tumorigenesis, but their significance as prognostic markers of BCR could not be validated.
42	Xu et al., 2015 [95]	Study of the role of miR-146-5p as a modulator of apoptosis in PCa cells by targeting ROCK1 based on the re-analysis the Taylor data set with 98 pat. [57]. BCR: PSA ≥0.2 µg/L on two occasions according to Taylor et al. [57].	Fresh-frozen tissue	Microarray (Agilent).	miR-146a-5p ↓	KMA of RFS: pat. with low level of miR-146a had shorter RFS than pat. with high level, log rank test, *p* < 0.048.	Low level of miR-146a represented a high BCR risk but multivariate analysis was not performed. The BCR analysis was obviously only intended to support the results of cell line experiments.
43	Bakkar et al., 2016 [96]	Discovery: in ERG differentially expressed PCa samples, miR-338-3p was identified as one of 11 differentially expressed miRs. Validation: miR-338-3p expression in 25 matched non-malignant vs. malignant PCa samples and RFS validation of this miR in the Taylor data set [57]. BCR: PSA ≥0.2 µg/L according to Taylor et al. [57].	FFPE	Discovery: microarray/RT-qPCR (TaqMan), RM: RNU48. Validation: microarray (Agilent) according to Taylor et al. [57].	miR-338-3p ↓	KMA of RFS: log-rank test, HR = 0.78, *p* = 0.02.	Less informative data regarding the usefulness of this miR for BCR prediction.
44	Bucay et al., 2016 [97]	Discovery/background: Based on the frequently genomic loss of chromosome 8p21 region in PCa and its association with the corresponding miR cluster, miR-3622b was examined as relevant cancer. Validation: 35 BCR vs. 57 non-BCR pat., follow-up up to ten years. BCR: PSA criterion not indicated.	FFPE	RT-qPCR (TaqMan); RM: RNU48.	miR-3622b-3p ↓	KMA: low miR-3622 expression predicted a shorter RFS, log rank test, *p* = 0.0321.	Low miR expression resulted in reduced BCR-free survival probability. Lack of evidence as independent factor because of the missing adjustment to standard clinical factors strongly limits the clinical significance.
45	Das et al., 2016 [98]	No background was given why miR-1207-3p was selected as a potential BCR marker. Study of RP specimens in 155 BCR vs. 249 non-BCR pat. BCR: PSA criterion not indicated.	FFPE	RT-qPCR (SYBR Green); RM: RNU6.	miR-1207-3p ↑	(1). miR expression higher in BCR pat. in comparison to non-BCR pat. (*t*-test, *p* < 0.0001). (2). CoxM: HR = 2.5, *p* < 0.001, adjusted for age and tumor stage.	PCa patients with a high miR-1207-3p expression had a high-risk of BCR.
46	Kristensen et al., 2016 [50]	Discovery: Training cohort 1 with RP specimens of localized PCa from 57 BCR vs. 69 non-BCR pat., mean follow-up 3 years. Validation: using 2 cohorts, own cohort 2 with 50 BCR vs. 60 non-BCR pat, mean follow-up 3.3; external cohort 3 of a publicly data set with 25 BCR vs. 74 non-BCR pat., follow-up 6 years. BCR: postoperative PSA >0.2 µg/L.	cohort 1 & 2: FFPE cohort 3: fresh-frozen tissue	For cohort 1 and 2: RT-qPCR platforms with different panels (Exiqon); RM: miR-151a-5p. Cohort 3: Microarray (Agilent).	RFS classifier: miR-185-5p ↑ miR-221-3p ↓ miR-326 ↓	(1). Development of a 3-BCR classifier from 11 individual miRs that remained significant in a multivariate model with standard clinicopathological factors. (2). KMA for RFS: log rank test, *p* < 0.050 in all 3 cohorts. (3). CoxM: Addition of the classifier to a multivariate model with clinicopathological factors increased the predictive accuracy.	This classifier (miR-185-5p + miR-221-3p + miR-326) was validated in two independent cohorts in an extensive manner and resulted in a benefit if included in a standard model with only clinicopathological factors.
47	Ling et al., 2016 [99]	Part of the study on the role of miR-30c and its target BCL9 in PCa progression and their combined use for BCR prediction: 18 BCR pat. vs. 80 non-BCR pat., median follow-up 3.8 years. These 98 pat. were identical to 98 pat. of 103 pat. included in a previous study about miR-30c [78]. BCR: postoperative PSA >0.2 µg/L.	Fresh-frozen tissue	RT-qPCR (GeneCopoeia); RM: RNU6B.	miR-30c-5p ↓ combined with its target BCL9	CoxU and CoxM: HR = 5.79 and 5.08, *p* = 0.023 and 0.048 for miR-30c/BCL9 status. This score remained an independent factor in the multivariate mode, together with the Gleason score.	The combined analysis of miR-30c and BCL9 may be a valuable tool for BCR prediction. The benefit of this score compared with miR-30c expression as shown in the previous study of the authors was not explained.
48	Nam et al., 2016 [100]	Based on a previous study about a 5-miR signature for BCR prediction [89], a more detailed study was performed using the single miR-301a: 585 PCa pat. (197 with BCR and 32 with metastasis vs, 388 non-BCR), median follow-up 8.4 years. BCR: PSA ≥0.2 µg/L on 2 consecutive follow-up visits that are at least 3 months apart.	FFPE	RT-qPCR (Qiagen); RM: miR-28-5p.	miR-301a-3p ↑	(1). No associations of miR-301a expression with conventional prognostic factors. (2). CoxU and CoxM: High level of miR-301a: HR = 1.55 and 1.42, *p* = 0.003 and *p* = 0.019. miR-301a remained an independent factor in the multivariate model, together with all conventional factors.	miR-301a may serve as a useful single BCR biomarker in combination with clinicopathological data. Illuminating mechanistic experiments regarding the role of miR-301a, but the authors did not comment whether this single miR could replace the 5-miR-signature recommend in their previous paper [89].
49	Nip et al., 2016 [101]	Discovery/background: based on a previous study on PCa cell lines that miR-4534 was upregulated [62]. Validation: 84 malignant vs. non-malignant matched PCa tissue samples, 34 BCR vs. 37 non-BCR., follow-up not given. BCR: PSA criterion not indicated.	Fresh-frozen tissue	RT-qPCR (TaqMan); RM: not defined.	miR-4534 ↑	KMA: high miR-4534 expression predicted a shorter RFS, log rank test, *p* < 0.01.	High miR-4534 expression was related to higher BCR risk. Lack of evidence of the miR as an independent factor because of the missing adjustment to standard clinical factors.
50	Xu et al., 2016 [102]	Discovery/background: miR-129 was examined in this study based on the role of miR-129 in other cancers [103]. Validation: 29 BCR vs. 89 non-BCR pat. BCR: PSA ≥0.2 µg/L following surgical treatment.	FFPE	RT-qPCR (Takara); RM: RNU6.	miR-129-5p ↓	(1). KMA: low miR-129 expression predicted a shorter RFS, log rank test, *p* < 0.001. (2). CoxU and CoxM: HR = 5.63 and 2.69, *p* < 0.001 in each case. miR-129 retained with the Gleason score, tumor and lymph node status as independent factors in the multivariate model.	Downregulation of miR-129 was associated with poor BCR-free survival.
51	Colden et al., 2017 [104]	Discovery/background: miR-466 was examined based on a previous study its downregulation PCa cell lines [62]. Validation: 92 PCa pat. from two sources, 34 BCR vs. 37 non-BCR pat., follow-up up to 12 years. BCR: first postoperative PSA >0.1 µg/L.	FFPE	RT-qPCR (TaqMan); RM: not defined.	miR-466 ↓	(1). Association of down-regulated miR-466 with the Gleason score, tumor stage (*p* < 0.0001). (2). KMA: low miR-466 expression predicted a shorter RFS, log rank test, *p* = 0.01. (3). Missing multivariate analysis.	Low expression of miR-466 can predict BCR.
52	Lin et al., 2017 [105]	Discovery/background: miR-30d was examined based on controversial expression and functional data [59,106]. Validation: with the Taylor data set [57] and TCGA data with 27 and 59 BCR and 80 and 365 non-BCR, respectively, follow-up up to 14 years. BCR: PSA ≥0.2 µg/L on two occasions after RP according to Taylor et al. [57].	Fresh-frozen tissue	Microarray (Agilent), see Taylor et al. [57].	Model with miR-30d-5p ↑ + MYPT1 ↓	(1). Upregulation of miR-30d and downregulation of its target MYPT1. (2). KMA: Combination of both (miR-30d^high^/MYPT1^low^) predicted better shorter RFS than markers alone (*p* = 0.003). (3). CoxM: HR = 5.13, *p* = 0.026, remained as an independent factor with tumor stage in the Taylor data set but not in the TCGA data set.	miR-30d/MYPT1 combination was identified as an independent factor to predict BCR of PCa patients, but controversial results in two data sets were shown.
53	Wei et al., 2017 [107]	Discovery/background: miR-1 was examined based on a previous study with miR-1 downregulation in recurrent cases [74]. Validation: 27 BCR vs. 51 non-BCR pat. of clinically localized PCa, follow-up within 4 years after RP. Recurrence definition: BCR with PSA <0.2 µg/L, local and systemic recurrence and cancer-related death.	FFPE	RT-qPCR (TaqMan); RM: RNU43.	miR-1-3p ↓	(1). Downregulated miR-1 in recurrent pat., (*t* test, *p* < 0.001). (2). ROC for recurrence: AUC = 0.885, *p* < 0.001. (3). CoxU and CoxM: HR = 1.53 and 1.86, *p* = 0.024 and *p* = 0.011.	miR-1 can function as an independent recurrence predictor together with standard clinicopathological variables.

^1^ Development phases are explained in Table 2. ^2^ Manufacturer/assay name is given in parentheses with the reference method (RM) in the validation process. ^3^ Significant ↓, downregulated and ↑, upregulated miRNAs predict a higher BCR risk. (-) indicates “not associated with BCR risk”. All miRs are adapted to the currently valid miRBase hsa-miR nomenclature, version 21. The miRBase Accession Numbers and the mature sequences of the miRNAs as truly stable identifiers are compiled in Supplementary Information, Supporting Table S1. APLN, Apelin; AUC, area under the ROC curve; BCL9, B-cell CLL/lymphoma 9; BCR, biochemical recurrence; BPH, benign prostatic hyperplasia; CoxU and CoxM, univariate and multivariate Cox regression analysis; FFPE; formalin-fixed, paraffin-embedded tissue; high-risk, PSA ≥ 20 µg/L and/or biopsy Gleason score ≥ 8 and/or clinical stage ≥ T3; HR, hazard ratio; KMA, Kaplan-Meier analysis; MW test, Mann-Whitney *U*-test; MYPT1, protein phosphatase 1 regulatory subunit 12A; pat., patients; PCa, prostate carcinoma; PSA, prostate-specific antigen; RB1CC1, RB1 inducible coiled-coil 1; RFS, biochemical recurrence-free survival; RM, reference method, in general the reference gene; ROC, receiver-operating characteristic curve; ROCK1, rho associated coiled-coil containing protein kinase 1; RP, radical prostatectomy; RT-qPCR, reverse transcription-quantitative polymerase chain reaction; SOCS, cytokine inducible SH2 containing protein; TRIB1, Tribbles pseudokinase 1.

**Table 4:** Distinct miRNAs analyzed in at least two studies for predicting biochemical recurrence.

miRNA	Studies, *n*	Study Nos. (Table 3)	References
miR-221-3p	6	↓: 3, 31, 46 ^a^; (-): 9, 14	[24,50,58,64,83]
miR-21-5p	4	↑: 11, 29; ↓:14, 31	[60,64,81,83]
miR-145-5p	4	↑: 5; ↓: 15, 17; (-): 9	[52,58,65,67]
miR-1-3p	3	↓: 8, 24, 53	[55,74,107]
miR-96-5p	3	↑: 2, 17; (-): 9	[23,58,67]
miR-30c-5p	2	↓: 28, 47	[78,99]
miR-30d-5p	2	↑: 10, 52	[59,105]
miR-133b	2	↑: 26; ↓:24	[74,76]
miR-141-3p	2	↓: 31, 36	[83,88]
miR-185-5p	2	↑: 46 ^a^	[50]
miR-195-5p	2	↓: 33, 34	[85,86]
miR-224-5p	2	↓: 27, 41	[77,94]
miR-301a-3p	2	↑: 37, 48	[89,100]
miR-326	2	↑: 46 ^a^	[50]
miR-182-5p	2	↑: 23; (-): 40	[49,93]

^a^ External validation was considered as a separate study. ↑, upregulated and ↓, downregulated miRNAs in the cohort with the higher BCR risk. (-) indicates “not associated with BCR risk”. The complete list of the 53 studies is given in Supplementary Information, Supporting Table S2.

**Table 5:** Characteristics of the 53 studies evaluated in this review.

Characteristics	Studies, *n* (%)
1. PSA cutoff for biochemical recurrence	
≥0.1 µg/L	4 (7)
≥0.2 µg/L	35 (66)
≥0.4 µg/L	2 (4)
Not specified	12 (23)
2. Preoperative PSA level	
<10 µg/L	3 (6)
>10 µg/L	43 (81)
Not specified	7 (13)
3. Tumor characteristics	
pT classification/clinical stage	
Specified	50 (94)
Not specified	3 (6)
Gleason score	
Specified	52 (98)
Not specified	1 (2)
Resection margin status	
Specified	21 (40)
Not specified	32 (60)
Lymph node status/Metastasis	
Specified	16 (30)
Not specified	37 (70)
4. Study design features	
According to MIQE, REMARK, STARD ^1^ guidelines	
Yes	2 (4)
No	51 (96)
Type of study	
Retrospective	53 (100)
Multi-institutional study (*n* ≥ 2)	9 (17)
Studies with functional miR data	
Yes	30 (57)
No	23 (43)
Sample size (patients/study)	
<50	7 (13)
50–100	23 (44)
>100–150	15 (28)
>150	8 (15)
Events of biochemical recurrence (n/study)	
10–20	11 (21)
20–30	15 (28)
>30	17 (32)
Not specified	10 (19)
Follow-up time (mean/median years)	
<5	18 (34)
>5	25 (47)
Not specified	9 (19)
Statistical analysis	
Only univariate	18 (34)
Multivariate	35 (66)
Studies with internal/external validation	
Yes	8 (15)
No	45 (85)

^1^ Reports with comments that the respective study was performed according to the of guidelines of MIQE, Minimum information for publication of quantitative real-time PCR experiments, REMARK, Reporting Recommendations for Tumor Marker Prognostic Studies , and/or STARD, Standards for Reporting of Diagnostic Accuracy [129,131,132].

## Supplementary Materials

Supplementary materials can be found at www.mdpi.com/1422-0067/18/10/2023/s1.

## Abbreviations

APLN	Apelin
AUA	American Urological Association
AUC	Area under the ROC curve
BCL9	B-cell CLL/lymphoma 9
BCR	Biochemical recurrence
BPH	Benign prostatic hyperplasia
CoxM	Multivariate Cox regression analysis
CoxU	Univariate Cox regression analysis
EAU	European Association of Urology
FFPE	Formalin-fixed, paraffin-embedded
HR	Hazard ratio
ISUP	International Society of Urological Pathology
KMA	Kaplan-Meier analysis
MeSH	Medical Subject Heading of the U.S. National Library of Medicine
miQ	miRNA index quote
MIQE	Minimum information for publication of quantitative real-time PCR experiments
miRNA, miR	microRNA
MW	Mann-Whitney U-test
MYPT1	Protein phosphatase 1 regulatory subunit 12A (official symbol: PPP1R12A)
PCa	Prostate carcinoma
PSA	Prostate-specific antigen
RB1CC1	RB1 inducible coiled-coil 1
REMARK	Reporting Recommendations for Tumor Marker Prognostic Studies
RFS	Biochemical recurrence-free survival
RM	Reference method, in general the reference gene
ROC	Receiver-operating characteristic curve
ROCK1	Rho associated coiled-coil containing protein kinase 1
RP	Radical prostatectomy
RT-qPCR	Reverse transcription-quantitative polymerase chain reaction
SOCS	Cytokine inducible SH2 containing protein (official symbol: CISH)
STARD	Standards for Reporting of Diagnostic Accuracy
TCGA	The Cancer Genome Atlas
TNM	Classification of malignant tumors describing the involment of the primary tumor, regional lymph nodes and the distant metastatic spread
TRIB1	Tribbles pseudokinase 1
