# Supplementary material for: Tissue-Based MicroRNAs as Predictors of Biochemical Recurrence after Radical Prostatectomy: What Can We Learn from Past Studies?
Source: Int J Mol Sci. 2017 Sep 21;18(10):2023. doi: 10.3390/ijms18102023 (PMC5666705; doi:10.3390/ijms18102023)
Supplement: Supplementary file 1 [file ijms-18-02023-s001.pdf]

Supplementary Material: Tissue-based MicroRNAs as Predictors of Biochemical Recurrence after Radical Prostatectomy: What Can We Learn from the Past Studies?

Zhongwei Zhao, Carsten Stephan, Sabine Weickmann, Monika Jung, Glen Kristiansen and Klaus Jung

Supplementary Table S1. microRNA-Identifiers of the microRNAs measured in the reviewed studies.

Study No.	Reference, Year	Mirna in the Study	Mirbase id (v21)	Mirbase Accession Number	Mature Sequence
1	Tong et al., 2009 [22]	miR-135b-5p<br>miR-194-5p	hsa-miR-135b-5p<br>hsa-miR-194-5p	MIMAT0000758<br>MIMAT0000460	uauggcuuuucauuccuauguga<br>uguaacagcaacuccaugugga
2	Schaefer et al., 2010 [23]	miR-96-5p	hsa-miR-96-5p	MIMAT0000095	uuuggcacuagcacauuuuugcu
3	Spahn et al., 2010 [24]	miR-221-3p	hsa-miR-221-3p	MIMAT0000278	agcuacauugucugcuggguuuc
4	Fendler et al., 2011 [25]	miR-10b-5p	hsa-miR-10b-5p	MIMAT0000254	uacccuguagaaccgaauuugug
5	Leite et al., 2011 [52]	miR-100-5p<br>miR-145-5p<br>miR-191-5p<br>let-7c-5p	hsa-miR-100-5p<br>hsa-miR-145-5p<br>hsa-miR-191-5p<br>hsa-let-7c-5p	MIMAT0000098<br>MIMAT0000437<br>MIMAT0000440<br>MIMAT0000064	aacccguagauccgaacuugug<br>guccaguuuucccaggaaucccu<br>caacggaaucccaaaagcagcug<br>ugagguaguagguuguaugguu
6	Long et al., 2011 [53]	miR-647<br>miR-519	hsa-miR-647	MIMAT0003317	guggcugcacucacuuccuuc
7	Barron et al., 2012 [54]	miR-200a-3p	hsa-miR-200a-3p	MIMAT0000682	uaacacugucugguaacgaugu
8	Hudson et al., 2012 [55]	miR-1-3p	hsa-miR-1-3p	MIMAT0000416	uggaauguaaagaaguauguau
9	Kang et al., 2012 [58]	miR-96-5p-5p<br>miR-145-5p<br>miR- 221-3p	hsa-miR-96-5p<br>hsa-miR-145-5p<br>hsa-miR-221-3p	MIMAT0000095<br>MIMAT0000437<br>MIMAT0000278	uuuggcacuagcacauuuuugcu<br>guccaguuuucccaggaaucccu<br>agcuacauugucugcuggguuuc
10	Kobayashi et al., 2012 [59]	miR-30d-5p	hsa-miR-30d-5p	MIMAT0000245	uguaaacauccccgacuggaag
11	Li et al., 2012 [60]	miR-21-5p	hsa-miR-21-5p	MIMAT0000076	uagcuuaucagacugauguuga
12	Majid et al., 2012 [62]	miR-23b-3p	hsa-miR-23b-3p	MIMAT0000418	aucacauugccagggauuacc
13	Saini et al., 2012 [63]	miR-708-5p	hsa-miR-708-5p	MIMAT0004926	aaggagcuuacaaucuagcuggg
14	Amank-wah et al., 2013 [64]	miR-21-5p<br>miR-221-3p<br>miR-222-3p	hsa-miR-21-5p<br>hsa-miR-221-3p<br>hsa-miR-222-3p	MIMAT0000076<br>MIMAT0000278<br>MIMAT0000279	uagcuuaucagacugauguuga<br>agcuacauugucugcuggguuuc<br>agcuacaucuggcuacugggu
15	Avgeris et al., 2013 [65]	miR-145-5p	hsa-miR-145-5p	MIMAT0000437	guccaguuuucccaggaaucccu
16	He et al., 2013 [66]	miR-374b-5p	hsa-miR-374b-5p	MIMAT0004955	auauaauacaaccugcuaagug
17	Larne et al., 2013 [67]	miR-96-5p<br>miR-145-5p<br>miR-183-5p<br>miR-221-5p	hsa-miR-96-5p<br>hsa-miR-145-5p<br>hsa-miR-183-5p<br>hsa-miR-221-5p	MIMAT0000095<br>MIMAT0000437<br>MIMAT0000261<br>MIMAT0004568	uuuggcacuagcacauuuuugcu<br>guccaguuuucccaggaaucccu<br>uauggcacugguagaauucacu<br>accuggcauacaauguagauuu
18	Lichner et al., 2013 [69]	miR-152-3p<br>miR-331-3p	hsa-miR-152-3p<br>hsa-miR-331-3p	MIMAT0000438<br>MIMAT0000760	ucagugcaugacagaacuugg<br>gccccugggccuauccuagaa
19	Majid et al., 2013 [70]	miR-34b-3p	hsa-miR-34b-3p	MIMAT0004676	caaucacuaacuccacugccau
20	Schubert et al., 2013 [71]	let-7b-5p	hsa-let-7b-5p	MIMAT0000063	ugagguaguagguugugugguu

Study No.	Reference, Year	Mirna in the Study	Mirbase id (v21)	Mirbase Accession Number	Mature Sequence
21	Sun et al., 2013 [72]	miR-126-3p	hsa-miR-126-3p	MIMAT0000445	ucguaccgugaguaauaaugcg
22	Avgeris et al., 2014 [73]	miR-378a-3p	hsa-miR-378a-3p	MIMAT0000732	acuggacuuggagucagaaggc
23	Casanova-Salas et al., 2014 [49]	miR-182-5p<br>miR-187-3p	hsa-miR-182-5p<br>hsa-miR-187-3p	MIMAT0000259<br>MIMAT0000262	uuuggcaaugguagaacucacacu<br>ucgugucuuguguugcagccgg
24	Karatas et al., 2014 [74]	miR-1-3p<br>miR-133b	hsa-miR-1-3p<br>hsa-miR-133b	MIMAT0000416<br>MIMAT0000770	uggaauguaaagaaguauguau<br>uuugguccccuucaaccagcua
25	Katz et al., 2014 [75]	miR-200b-3p	hsa-miR-200b-3p	MIMAT0000318	uaauacugccugguaaugauga
26	Li et al., 2014 [76]	miR-133b	hsa-miR-133b	MIMAT0000770	uuugguccccuucaaccagcua
27	Lin et al., 2014 [77]	miR-224-5p	hsa-miR-224-5p	MIMAT0000281	caagucacuagugguuccguu
28	Ling et al., 2014 [78]	miR-30c-5p	hsa-miR-30c-5p	MIMAT0000244	uguaaacauccuacacucucagc
29	Melbø-Jørgensen et al., 2014 [81]	miR-21-5p	hsa-miR-21-5p	MIMAT0000076	uagcuuaucagacugauguuga
30	Mortensen et al., 2014 [82]	miR-449b-5p	hsa-miR-449b-5p	MIMAT0003327	aggcaguguauuguuagcuggc
31	Zheng et al., 2014 [83]	miR-21-5p<br>miR-141-3p<br>miR-221-3p	hsa-miR-21-5p<br>hsa-miR-141-3p<br>hsa-miR-221-3p	MIMAT0000076<br>MIMAT0000432<br>MIMAT0000278	uagcuuaucagacugauguuga<br>uaacacugucugguaaagaugg<br>agcuacauugucugcuggguuuc
32	Bell et al., 2015 [84]	miR-4516<br>miR-601	hsa-miR-4516<br>hsa-miR-601	MIMAT0019053<br>MIMAT0003269	gggagaagggucggggc<br>uggucuaggauuguuggaggag
33	Cai et al., 2015 [85]	miR-195-5p	hsa-miR-195-5p	MIMAT0000461	uagcagcacagaaauauuggc
34	Guo et al., 2015 [86]	miR-195-5p	hsa-miR-195-5p	MIMAT0000461	uagcagcacagaaauauuggc
35	Leite et al., 2015 [87]	miR-21-3p	hsa-miR-21-3p	MIMAT0004494	caacaccagucgaugggcugu
36	Lichner et al., 2015 [88]	miR-29c-3p<br>miR-141-3p<br>miR-148a-3p<br>miR-34a-5p	hsa-miR-29c-3p<br>hsa-miR-141-3p<br>hsa-miR-148a-3p<br>hsa-miR-34a-5p	MIMAT0000681<br>MIMAT0000432<br>MIMAT0000243<br>MIMAT0000255	uagcaccauuugaaaucgguua<br>uaacacugucugguaaagaugg<br>ucagugcacuacagaacuuugu<br>uggcagugucuuagcugguugu
37	Nam et. al 2015 [89]	miR-301a-3p<br>miR-652-3p<br>miR-454-3p<br>miR-223-3p<br>miR-139-5p	hsa-miR-301a-3p<br>hsa-miR-652-3p<br>hsa-miR-454-3p<br>hsa-miR-223-3p<br>hsa-miR-139-5p	MIMAT0000688<br>MIMAT0003322<br>MIMAT0003885<br>MIMAT0000280<br>MIMAT0000250	cagugcaauaguauugucaaagc<br>aauggcgccacuaggguugug<br>uagugcaauauugcuuauagggu<br>ugucaguuugucaaauacccca<br>ucuacagugcacgugucuccagu
38	Sun et al., 2015 [90]	miR-128-3p	hsa-miR-128-3p	MIMAT0000424	ucacagugaaccggucucuuu
39	Tian et al., 2015 [92]	let-7a-5p	hsa-let-7a-5p	MIMAT0000062	ugagguaguagguuguauaguu
40	Wallis et al., 2015 [93]	miR-182-5p	hsa-miR-182-5p	MIMAT0000259	uuuggcaaugguagaacucacacu
41	Wan et al., 2015 [94]	miR-224-5p	hsa-miR-224-5p	MIMAT0000281	caagucacuagugguuccguu
42	Xu et al., 2015 [95]	miR-146a-5p	hsa-miR-146a-5p	MIMAT0000449	ugagaacugaauuccauggguu
43	Bakkar et al., 2016 [96]	miR-338-3p	hsa-miR-338-3p	MIMAT0000763	uccagcaucagugauuuuguug
44	Bucay et al., 2016 [97]	miR-3622b-3p	hsa-miR-3622b-3p	MIMAT0018006	ucaccugagcuccccgugccug

Study No.	Reference, Year	Mirna in the Study	Mirbase id (v21)	Mirbase Accession Number	Mature Sequence
45	Das et al., 2016 [98]	miR-1207-3p	hsa-miR-1207-3p	MIMAT0005872	ucagcuggcccucauuuc
46	Kristensen et al., 2016 [50]	miR-185-5p<br>miR-221-3p<br>miR-326	hsa-miR-185-5p<br>hsa-miR-221-3p<br>hsa-miR-326	MIMAT0000455<br>MIMAT0000278<br>MIMAT0000756	uggagagaaaggcaguuccuga<br>agcuacauugucugcuggguuuc<br>ccucugggcccuuccuccag
47	Ling et al., 2016 [99]	miR-30c-5p	hsa-miR-30c-5p	MIMAT0000244	uguaaacauccuacacucucagc
48	Nam et al., 2016 [100]	miR-301a-3p	hsa-miR-301a-3p	MIMAT0000688	cagugcaauaguauugucaaagc
49	Nip et al., 2016 [101]	miR-4534	hsa-miR-4534	MIMAT0019073	ggauggaggaggggucu
50	Xu et al., 2016 [102]	miR-129-5p	hsa-miR-129-5p	MIMAT0000242	cuuuuugcggucugggcuugc
51	Colden et al., 2017 [104]	miR-466	hsa-miR-466	MIMAT0015002	auacacauacacgcaacacacau
52	Lin et al., 2017 [105]	miR-30d-5p	hsa-miR-30d-5p	MIMAT0000245	uguaaacauccccgacuggaag
53	Wei et al., 2017 [107]	miR-1-3p	hsa-miR-1-3p	MIMAT0000416	uggaauguaaagaaguauguau

Supplementary Table S2. Number of studies using distinct miRNAs for predicting biochemical recurrence.

Number of Studies	miRNA	Study no. in Table 3	References of the Main Text
6	miR-221-3p	↓: 3, 31, 46 [a]; (-):9, 14	[24,50,58,64,83]
4	miR-21-5p	↑: 11, 29; ↓:14, 31	[60,64,81,83]
4	miR-145-5p	↑: 5; ↓15, 17; (-):9	[52,58,65,67]
3	miR-1-3p	↓: 8, 24, 53	[55,74,107]
3	miR-96-5p	↑: 2, 17; (-):9	[23,58,67]
2	miR-30c-5p	↓: 28, 47	[78,99]
2	miR-30d-5p	↑: 10, 52	[59,105]
2	miR-133b	↑: 26; ↓:24	[74,76]
2	miR-141-3p	↓: 31, 36	[83,88]
2	miR-185-5p	↑: 46 [a]	[50]
2	miR-195-5p	↓: 33, 34	[85,86]
2	miR-224-5p	↓: 27, 41	[77,94]
2	miR-301a-3p	↑: 37, 48	[89,100]
2	miR-326	↑: 46 [a]	[50]
2	miR-182-5p	↑: 23; (-):40	[49,93]
1	let-7a-5p	↓: 39	[92]
1	let-7b-5p	↓: 20	[71]
1	let-7c-5p	↑: 5	[52]
1	miR-10b-5p	↑: 4	[25]
1	miR-21-3p	↑: 35	[87]
1	miR-23b-3p	↓: 12	[62]
1	miR-29c-3p	↓: 36	[88]
1	miR-34a-5p	↓: 36	[88]
1	miR-34b-3p	↓: 34	[86]
1	miR-100-5p	↑: 5	[52]
1	miR-126-3p	↓: 21	[72]
1	miR-128-3p	↓: 38	[90]
1	miR-129-5p	↓: 10	[59]
1	miR-135b-5p	↑: 1	[22]
1	miR-139-5p	↓: 37	[89]
1	miR-146a-5p	↓: 42	[95]
1	miR-148a-3p	↓: 36	[88]
1	miR-152-3p	↓: 18	[69]
1	miR-183-5p	↑: 17	[67]
1	miR-187-3p	↓: 23	[49]
1	miR-191-5p	↑: 5	[52]
1	miR-194-5p	↑: 1	[22]
1	miR-200a-3p	↓: 7	[54]
1	miR-200b-3p	↓: 25	[75]
1	miR-221-5p	↓: 17	[67]
1	miR-222-3p	(-) 14	[64]
1	miR-223-3p	↓: 37	[89]
1	miR-331-3p	↓: 18	[69]
1	miR-338-3p	↓: 43	[96]
1	miR-374b-5p	↓: 16	[66]
1	miR-378a-3p	↓: 22	[73]
1	miR-449b-5p	↑: 30	[82]
1	miR-454-3p	↑: 37	[89]
1	miR-466	↓: 51	[104]
1	miR-519	↑: 6	[53]
1	miR-601	↑: 32	[84]
1	miR-647	↓: 6	[53]
1	miR-652-3p	↑: 37	[89]

Number of Studies	miRNA	Study no. in Table 3	References of the Main Text
1	miR-708-5p	↓: 13	[63]
1	miR-1207-3p	↑: 45	[98]
1	miR-3622b-3p	↓: 44	[97]
1	miR-4516	↑: 32	[84]
1	miR-4534	↑: 49	[101]

[a] An external validation was considered as separate study. ↑, upregulated and ↓, downregulated miRNAs in the cohort with the higher BCR risk. (-) indicates "not associated with BCR risk".
